# Complete mitochondrial genome of the lemon damsel, *Pomacentrus moluccensis* (Perciformes, Pomacentridae)

**DOI:** 10.1080/23802359.2020.1768952

**Published:** 2020-05-22

**Authors:** Sang-Eun Nam, Jae-Sung Rhee

**Affiliations:** a Department of Marine Science, College of Natural Sciences, Incheon National University, Incheon, South Korea; b Research Institute of Basic Sciences, Incheon National University, Incheon, South Korea

**Keywords:** *Pomacentrus moluccensis*, lemon damsel, mitogenome, Pomacentrinae

## Abstract

*Pomacentrus moluccensis* Bleeker, 1853 (Pomacentridae) is a bright yellow damselfish. Here, we assembled a complete 16,801 bp genome for *P. moluccensis* mitochondrion by employing the Illumina MiSeq platform. The complete mitochondrial genome contained 13 protein-coding genes (PCGs), 22 transfer RNA (tRNA) genes, two ribosomal RNA (rRNA) genes, and one putative control region. The overall genomic structure of *P. moluccensis* mitochondrion was conserved with the gene arrangements of mitogenomes published in subfamily Pomacentrinae, and phylogenetic analysis confirmed the sister relationship among the genus *Amblyglyphidodon*, *Amphiprion*, and *Chrysiptera*. This complete mitochondrial DNA information of *P. moluccensis* will provide essential molecular reference to elucidate the geographical distribution, the phylogenetic relationship, and evolutionary history of the damselfish genus *Pomacentrus*.

The damselfish (Perciformes, Pomacentridae) are one of the most abundant and diverse families of coral reef fish taxa (Allen 1991). They are relatively small acanthopterygian fishes and are widely distributed with greatest diversity with crucial ecological positions on coral and rocky reef communities (Cooper et al. 2009). The subfamily Pomacentrinae is the most diversified of the five Pomacentridae subfamilies with complex phylogeographic and taxonomic history (Cooper et al. 2009). The genus *Pomacentrus* Lacepéde, 1802 comprises 77 species and is the second most diversified damselfish (Allen et al. 2017). Since *Pomacentrus* has contrasting geographic distribution, analysis of the phylogenetic relationship and diversification pattern has consistently been highlighted the genus as suitable models for the evolutionary diversification study (Drew and Barber 2009; Liu et al. 2012; Allen et al. 2017).

The coral-dwelling damselfish *Pomacentrus moluccensi*, also known as the lemon damsel, is widely distributed in the Indo-Pacific region (Drew and Barber 2009). In this study, we sequenced the complete mitogenome of *P. moluccensis* (Accession no. MT410992). An individual of *P. moluccensis* was sampled at East China Sea (30°45′33.0″N 126°22′10.2″E) in July 2013. The voucher specimen was deposited in the Research Institute of Basic Sciences of Incheon National University (Specimen ID: 2008-Pomacentridae025). Genomic DNA was extracted from muscle tissue by using the DNeasy Blood and Tissue kit (Qiagen, Hilden, Germany). Genomic DNA was quantified using a Qubit 4 Fluorometer (Thermo Fisher Scientific, Inc., Waltham, MA, USA). The library construction and Illumina MiSeq sequencing were performed by a commercial company (Phyzen, Seoul, South Korea). Genomic libraries were constructed from the total genomic DNA (1 μg) using the TruSeq RNA Sample Preparation Kit according to the manufacturer’s instructions (Illumina, San Diego, CA, USA). Additional PCR procedure was conducted to confirm the DNA sequence of the control region. Overall sequences were annotated using the MITOS web-based software (Bernt et al. 2013) and detailed annotation was conducted with NCBI-BLAST (http://blast.ncbi.nlm.nih.gov).

The complete mitogenome of *P. moluccensis* was 16,801 bp in length and contained a typical set of 13 PCGs, 22 tRNAs, two rRNAs, and one control region, as shown in Pomacentridae mitogenomes (Nam and Rhee 2020). Preliminary phylogenetic analysis using cytochrome c oxidase 1 (*CO1*) mitochondrial DNA sequence showed that *P. moluccensis* grouped together with other representatives of *Pomacentrus* species in the family Pomacentridae (Figure 1; small circular tree). A phylogenetic analysis was constructed using the concatenated set of 13 PCGs of *P. moluccensis* mitogenome with including 16 published mitogenomes from Pomacentridae and Mugilidae (outgroup) (Figure 1). We used JModelTest ver. 2.1.10 (Darriba et al. 2012) to select the best substitution model and a substitution model (HKY + G + I) was employed to construct a maximum-likelihood (ML) method in the PhyML 2.4.5 (Guindon and Gascuel 2003) with 1000 bootstrap replicates. The *P. moluccensis* mitogenome was clustered into subfamily Pomacentrinae. In conclusion, complete *P. moluccensis* mitogenome will provide useful dataset to elucidate the phylogenetic relationship, geographical distribution, and evolutionary history of the genus *Pomacentrus* and related subspecies.

**Figure 1.**
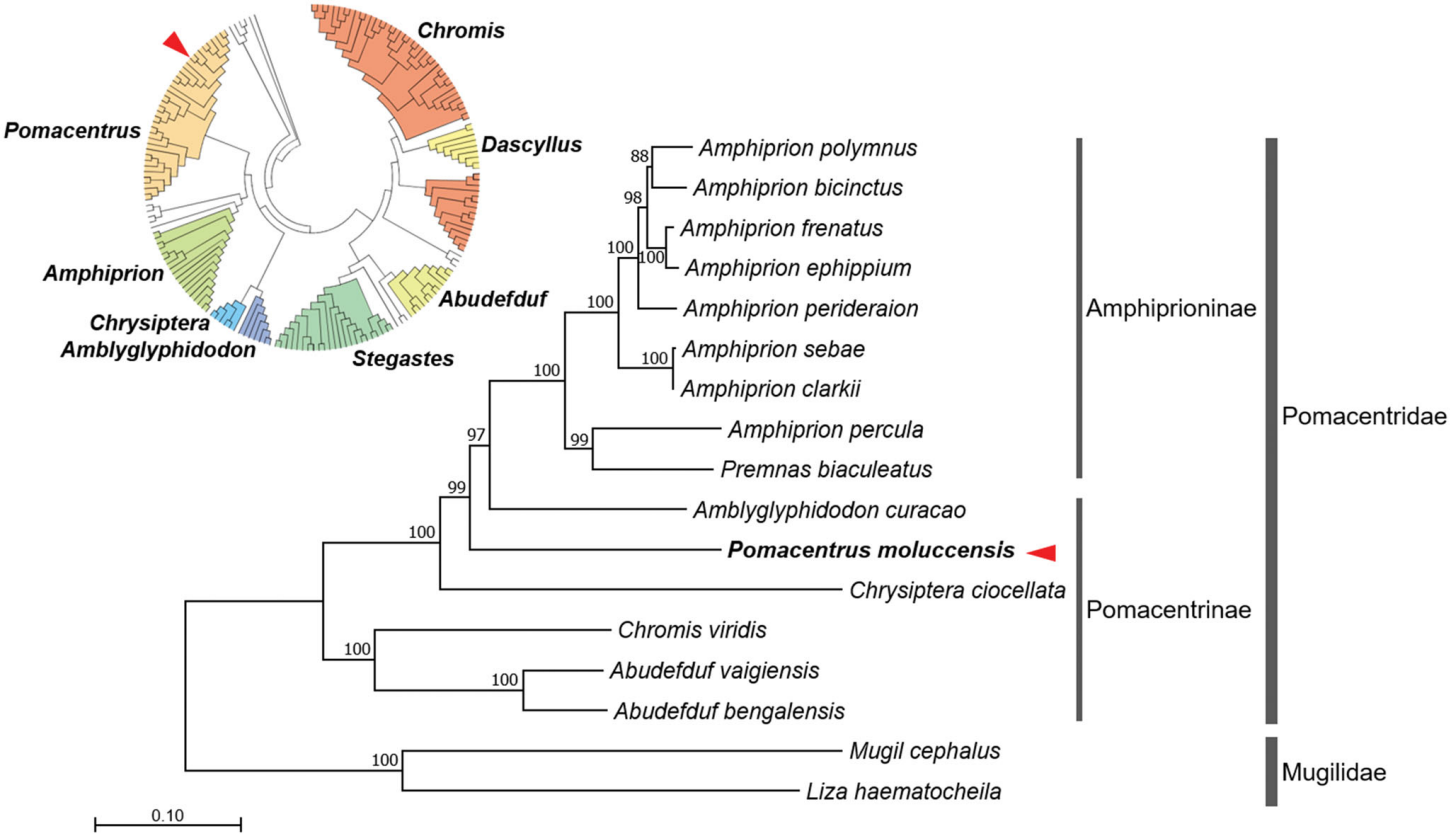
Maximum-likelihood (ML) phylogeny of 16 species of the family Pomacentridae based on the concatenated nucleotide sequences of entire protein-coding genes (PCGs). Two species from the family Mugilidae were used as outgroup. Numbers on the branches indicate ML bootstrap percentages (1000 replicates). DDBJ/EMBL/Genbank accession numbers for published sequences are incorporated. The red arrow indicates the *P. moluccensis* analyzed in this study.

## Data Availability

The data that support the findings of this study are openly available in National Center for Biotechnology Information (NCBI) at https://www.ncbi.nlm.nih.gov, accession number MT410992.
